# DHX15 is associated with poor prognosis in acute myeloid leukemia (AML) and regulates cell apoptosis via the NF-kB signaling pathway

**DOI:** 10.18632/oncotarget.20288

**Published:** 2017-08-16

**Authors:** Lili Pan, Yang Li, Hai-Ying Zhang, Yi Zheng, Xiao-Li Liu, Zheng Hu, Yi Wang, Jing Wang, Yuan-Hua Cai, Qiao Liu, Wan-Ling Chen, Ying Guo, Yuan-Mao Huang, Feng Qian, Li Jin, Jiucun Wang, Shao-Yuan Wang

**Affiliations:** ^1^ Fujian Institute of Hematology, Fujian Provincial Key Laboratory on Hematology, Department of Hematology, Fujian Medical University Union Hospital, Fuzhou, PR China; ^2^ Union Clinical Medical Colleges, Fujian Medical University, Fuzhou, PR China; ^3^ State Key Laboratory of Genetic Engineering and Ministry of Education Key Laboratory of Contemporary Anthropology, School of Life Sciences, Fudan University, Shanghai, PR China

**Keywords:** *DHX15*, AML, NF-kB, knockdown, overexpression

## Abstract

The role of *DHX15*, a newly identified DEAH-box RNA helicase, in leukemogenesis remains elusive. Here, we identified a recurrent mutation in *DHX15* (NM_001358:c.664C>G: p.(R222G)) in one familial AML patient and 4/240 sporadic AML patients. Additionally, *DHX15* was commonly overexpressed in AML patients and associated with poor overall survival (OS) (P=0.019) and relapse-free survival (RFS) (P=0.032). In addition, we found a distinct expression pattern of *DHX15*. *DHX15* was highly expressed in hematopoietic stem cells and leukemia cells but was lowly expressed in mature blood cells. *DHX15* was down-regulated when AML patients achieved disease remission or when leukemia cell lines were induced to differentiate. *DHX15* silencing greatly inhibited leukemia cell proliferation and induced cell apoptosis and G1-phase arrest. In contrast, the restoration of *DHX15* expression rescued cell viability and reduced cell apoptosis. In addition, we found that DHX15 was down-regulated when cell apoptosis was induced by ATO (arsenic trioxide); overexpression of DHX15 caused dramatic resistance to ATO-induced cell apoptosis, suggesting an important role for DHX15 in cell apoptosis. We further explored the mechanism of DHX15 in apoptosis and found that overexpression of *DHX15* activated *NF-kB* transcription. Knockdown of *DHX15* inhibited the nuclear translocation and activation of the NF-kB subunit P65 in leukemia cells. Several downstream targets of the NF-kB pathway were also down-regulated, and apoptosis-associated genes *CASP3* and *PARP* were activated. In conclusion, this study represents the first demonstration that *DHX15* plays an important role in leukemogenesis via the NF-kB signaling pathway and may serve as an independent prognostic marker for AML.

## INTRODUCTION

RNA helicases are ubiquitous, highly conserved enzymes that participate in nearly all aspects of RNA metabolism, including transcription, pre-mRNA splicing, mRNA export, ribosome biogenesis, translation and RNA degradation. Based on the conserved sequence motif required for ATPase activity, RNA helicases are classified into three subfamilies: DEAD-, DEAH- and DExH-box proteins.[1]

RNA helicases have received significant attention since their identification in the 1980s. Many RNA helicases are essential for cell viability, and a growing number of these enzymes have been implicated in carcinogenesis. RNA helicase *DHX29* is overexpressed in various types of cancers,[2] and the down-regulation of *DHX29* leads to impaired translation and suppression of cancer cell growth *ex vivo* and *in vivo*.[2] *DDX5* is highly expressed in primary human T-ALL leukemia cells.[3] Knockdown of *DDX5* results in reduced cell proliferation and increased apoptosis in cultured human leukemia cells and suppression of growth of human leukemia xenografts in nude mice.[3]

*DHX15*, a new member of the DEAH-box RNA helicase family, has been shown to localize in the nucleus and participate in modulating pre-mRNA splicing with its helicase activities.[4, 5] *DHX15* has been reported to be ubiquitously expressed in several tumor cell lines and multiple normal tissues and organs,[6] although its levels of expression vary. DNA sequence copy number gains of *DHX15* have been found in 39% of Barrett's adenocarcinoma cases [7] and 80% of malignant peripheral nerve sheath tumors.[8] Down-regulation of *DHX15* greatly inhibits proliferation in breast cancer cells, and co-overexpression of *DHX15* and *GPATCH2* enhances breast cancer cell growth.[9] Several studies report an identical mutation of *DHX15* (R222G) in MDS and AML patients, particularly in AML patients with t(8; 21).[10–12] One study reported 6/85 (7%) patients with *RUNX1-RUNX1T1/* t(8; 21)-positive AML carrying the *DHX15* mutation (R222G) using whole-exome sequencing technology.[13] In addition, these authors prove that the R222G mutation leads to impaired pre-mRNA splicing and weakened interactions between *DHX15* and other splicing components such as TFIP11. In addition, a similar increase in the number of alternative splicing events is observed when *DHX15* is down-regulated. These studies suggest that mutations or aberrant expression of *DHX15* may contribute to carcinogenesis and leukemogenesis. However, the role of *DHX15* in leukemogenesis remains unknown.

Herein, we demonstrated the recurrence of a *DHX15* mutation (NM_001358:c.664C>G:p.(R222G)) in a familial AML patient and 4/240 sporadic AML patients. In addition, we further examined the expression profile of *DHX15* in AML and normal bone marrow, as well as the function and pathogenesis of *DHX15* in AML. We concluded that *DHX15* may contribute to leukemogenesis and would be a promising marker for AML diagnosis, prognosis and MRD detection.

## RESULTS

### DHX15 somatic mutation is recurrent in AML patients

**Figure 1:**
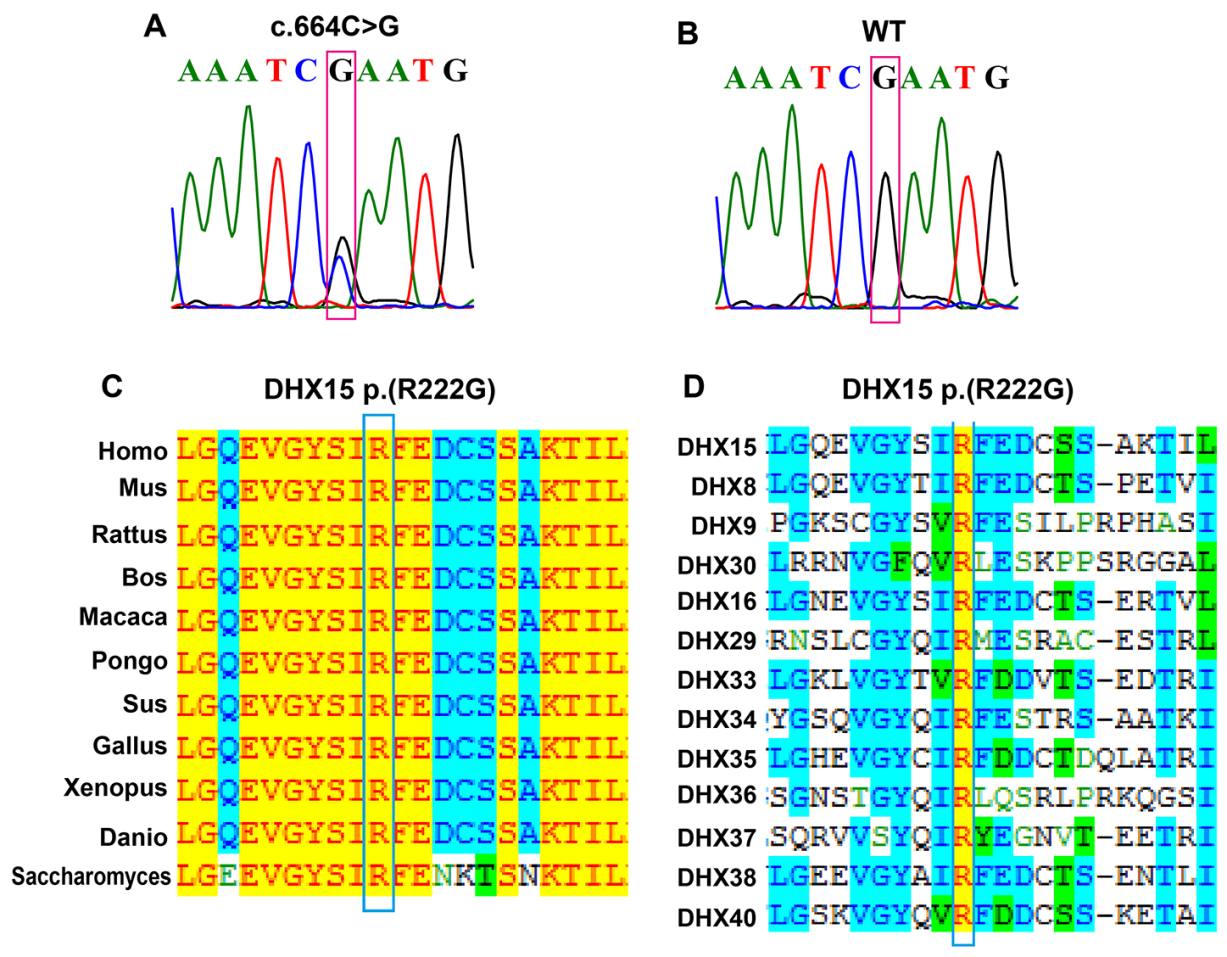
Identification of a *DHX15* somatic mutation in AML patients **(A)** Sanger sequencing of III-15 at his AML onset and 4 sporadic AML patients at their diagnosis confirmed the presence of a *DHX15* mutation (NM_001358:c.664C>G:p.(R222G)). **(B)** Sanger sequencing of III-15 before his AML onset and 4 *DHX15* mutation carrying patients after they achieved disease remission confirmed the absence of the *DHX15* mutation. **(C)** Alignment of *DHX15* amino acid sequences in 11 species, which suggests that the affected amino acid R222 was located at a highly conserved position during evolution. The left column represents the species, and the right shows the amino acid sequence in the corresponding species; amino acids that are identical to those in Homo sapiens are highlighted in yellow; those conservative to homo sapiens are highlighted in blue; and those weakly similar or non-similar are not highlighted. **(D)** Alignment of *DHX15* amino acid sequences in 13 members of the DEAH-box RNA helicase family, which suggests that the affected amino acid R222 was located at a highly conserved position.

We identified 13 somatic nonsynonymous mutations (Supplementary Table 2) in the familial AML patient (III-15) using whole exome sequencing (WES) followed by Sanger sequencing. The somatic mutations were further screened in 240 sporadic AML patients and 508 healthy controls using SNaPshot technology. We identified a recurrent mutation in *DHX15* (NM_001358:c.664C>G:p.(R222G)) that was present in 4/240 sporadic cases (Figure 1A). The mutation (R222G) disappeared when the affected patients achieved disease remission (Figure 1B). In addition, the mutation was absent in 508 healthy controls and the Exome Variant Server, 1000 Genome Project and dbSNP139 databases. When aligning the amino acid sequence between human and other 10 species (from mouse to yeast), we found that human *DHX15* was a highly conserved protein, sharing 99%, 83% and 80% identities of the amino acid sequence with mouse, zebrafish, and yeast, respectively. The mutation (R222G) was at a highly conserved position (Figure 1C and 1D).

### Overexpression of DHX15 is a common event in AML and associated with poor outcome

**Figure 2:**
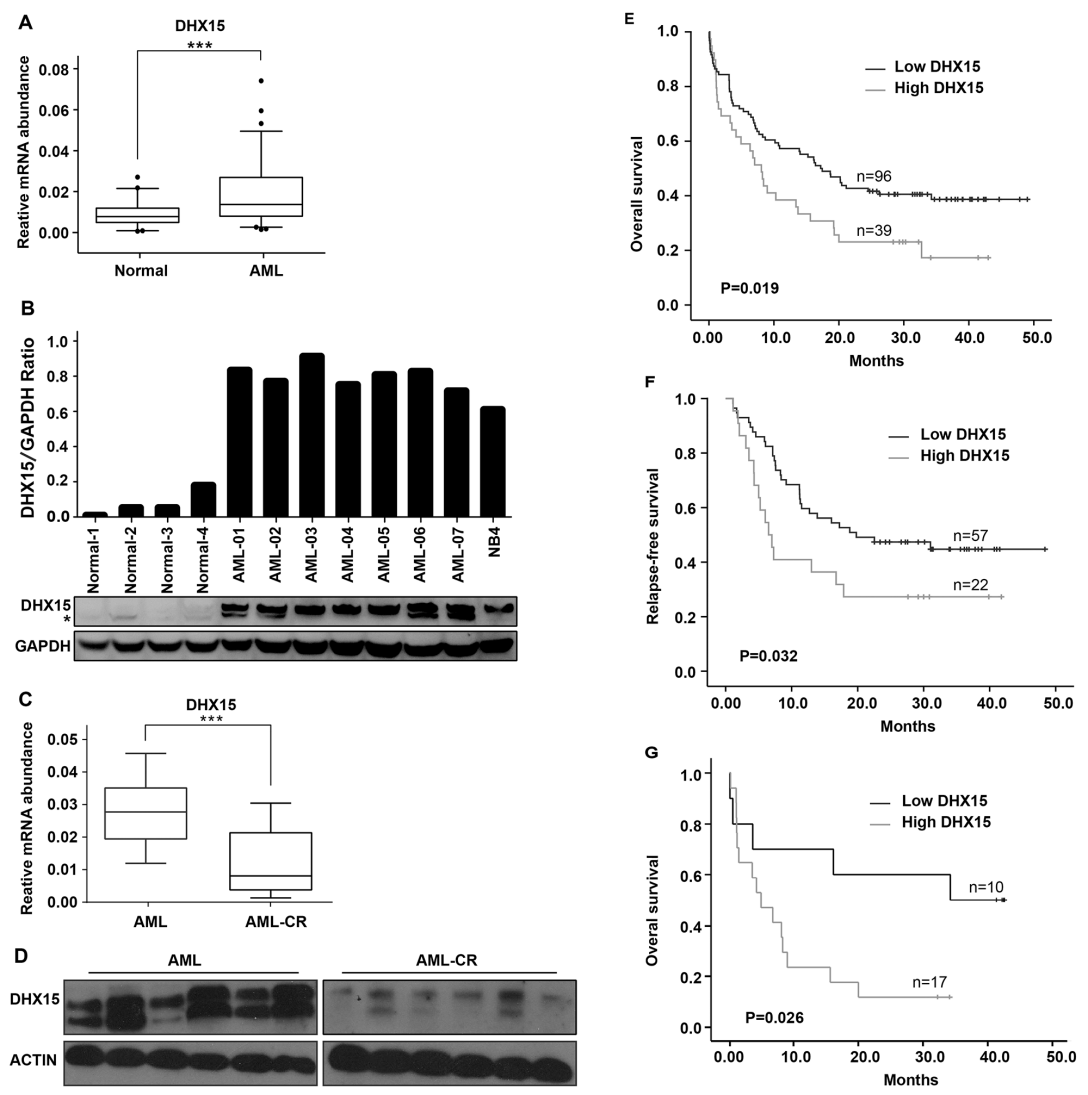
Prevalence and prognostic impact of *DHX15* overexpression in acute myeloid leukemia **(A)** Quantitative real-time PCR of the expression level of *DHX15* in 135 de novo AML patients and 84 normal controls. ^***^P<0.001 (B) Western blotting analysis of the expression level of *DHX15* in 7 AML patients and 4 normal controls. The samples are shown as representative examples. **(C)** qRT-PCR of *DHX15* in 30 paired samples from patients at their diagnosis of AML and disease remission phase. **(D)** Western blots of *DHX15* in 6 paired samples from patients at their diagnosis of AML and disease remission phase. **(E-F)** Kaplan-Meier analysis of overall survival (OS) and relapse-free survival (RFS) of AML patients with high or low expression levels of *DHX15*. **(G)** Overall survival of AML patients with high or low expression levels of *DHX15* in the group with poor cytogenetics.

The role of *DHX15* in leukemia remains elusive. We first quantified the expression of *DHX15* in 135 de novo AML patients and 84 normal controls by qRT-PCR and found that *DHX15* was overexpressed in 28.89% of cases (39/135) (Figure 2A). Similar to the mRNA expression pattern, the protein expression of *DHX15* via western blot was significantly increased in 7 AML patients compared to that of 4 healthy controls (Figure 2B). In addition, the expression of *DHX15* decreased when the patients who had overexpressed *DHX15* achieved disease remission (Figure 2C and 2D).

We analyzed the correlation between clinical characteristics and *DHX15* expression levels in AML patients and found that *DHX15* overexpression was associated with poor cytogenetic prognosis (P<0.001) (Table 1). The prevalence of *DHX15* overexpression in three cytogenetic prognostic groups was 0% (0/24) in the group with good cytogenetics, 26.19% (22/84) in the group with intermediate cytogenetics, and 62.96% (17/27) in the group with poor cytogenetics. There was no relationship between the *DHX15* expression level and CR rate (P=0.751). However, patients with *DHX15* overexpression had a significantly worse OS (P=0.019) (Figure 2E) and RFS (P=0.032) (Figure 2F). In the group with poor cytogenetics, the OS of patients with high *DHX15* expression was even worse (P=0.026) than that of patients with low *DHX15* expression (Figure 2G). Multivariate analysis demonstrated that *DHX15* overexpression was an unfavorable independent factor associated with OS in AML (P=0.018, HR=1.698,CI: 1.095-2.633).

**Table 1:** Clinical and molecular features of AML cases with or without DHX15 overexpression

	No. cases	DHX15− (%)	DHX15+ (%)	P
Mean age at diagnosis (years)		43.6±17.6	46.9±19.1	0.333
**Age**				
<60 years(%)	101	72(71.29)	29(28.71)	0.938
≥ 60 years(%)	34	24(70.59)	10(29.41)	
**Gender**				
Male (%)	73	54(73.97)	19(26.03)	0.426
Female (%)	62	42(67.74)	20(32.26)	
**WBC count**				
>10 × 109/L	107	75(70.09)	32(29.91)	0.610
<10 × 109/L	28	21(75)	7(25)	
**Cytogenetic group**				
Good	24	24(100)	0(0)	<0.001^*^
Intermediate	84	62(73.81)	22(26.19)	
Poor	27	10(37.04)	17(62.96)	
**FLT3-ITD**				
No(%)	90	63(70)	27(30)	0.687
Yes(%)	45	33(73.33)	12(26.67)	
**NPM1 mutated**				
No(%)	92	66(71.74)	26(28.26)	0.814
Yes(%)	43	30(69.77)	13(30.23)	
**CEBPA mutated**				
No(%)	123	87(70.73)	36(29.27)	0.756
Yes(%)	12	9(75)	3(25)	
**Complete remission**				
No(%)	56	39(69.64)	17(30.36)	0.751
Yes(%)	79	57(72.15)	22(27.85)	

### DHX15 may be associated with hematopoietic cell differentiation

**Figure 3:**
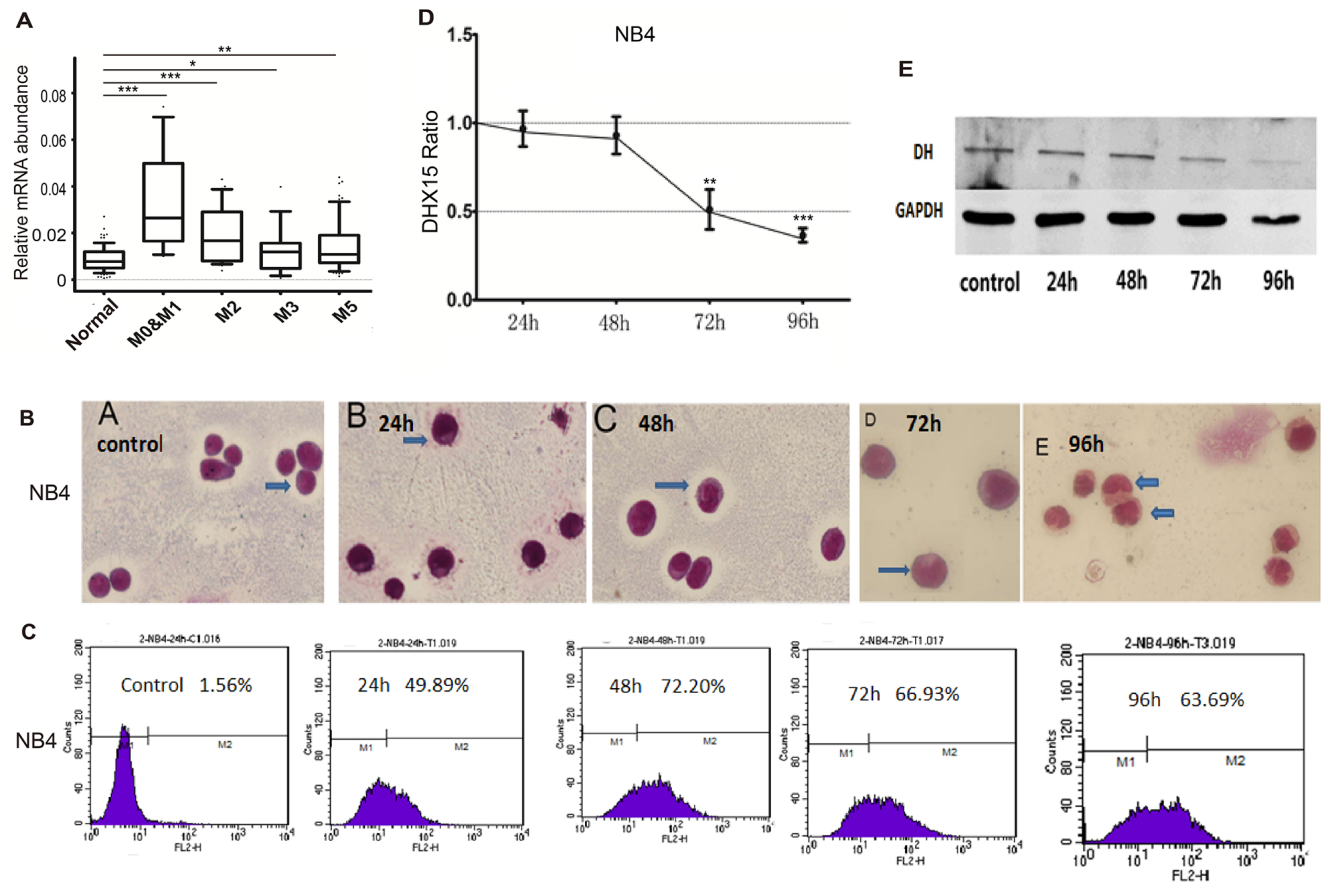
*DHX15* may be associated with hematopoietic cell differentiation **(A)** *DHX15* expression was higher in patients with subtypes M0, M1 and M2 than those with subtypes M3, M4 and M5. **(B-C)** NB4 cells showed differentiation-associated changes, including the formation of cytoplasmic neutrophil granules and increased expression of CD11b. **(D-E)** DHX15 expression was significantly decreased after being treated with ATRA for 72 or 96 h. ^*^P<0.05, ^**^P<0.01, ^***^P<0.001.

*DHX15* expression was higher in patients with subtypes M0, M1 and M2 than in those with subtypes M3, M4 and M5 (Figure 3A). In addition, *DHX15* expression was higher in the early phase of stem cells, including embryonic stem cells (ESCs) and hematopoietic stem cells (HSCs), and then appeared to decrease as the cells progressed through more mature stages (including common myeloid progenitors (CMPs) and granulocyte and macrophage progenitors (GMPs)), reaching its lowest level in granulocytes and macrophages (data from the EMBL database, see Supplementary Figure 2). The distinctive expression pattern of *DHX15* suggested its role in the regulation of myeloid differentiation; we, therefore, detected the expression level of *DHX15* during differentiation induced by all-trans-retinoic acid (ATRA) in human acute promyelocytic leukemia cells (NB4 cells) (Figure 3B-3E). In addition, we found that *DHX15* expression in leukemia cells was significantly decreased in a time-dependent manner with cell differentiation.

### Silencing DHX15 inhibits leukemia cell proliferation, and restoration of DHX15 expression restores cell proliferation

**Figure 4:**
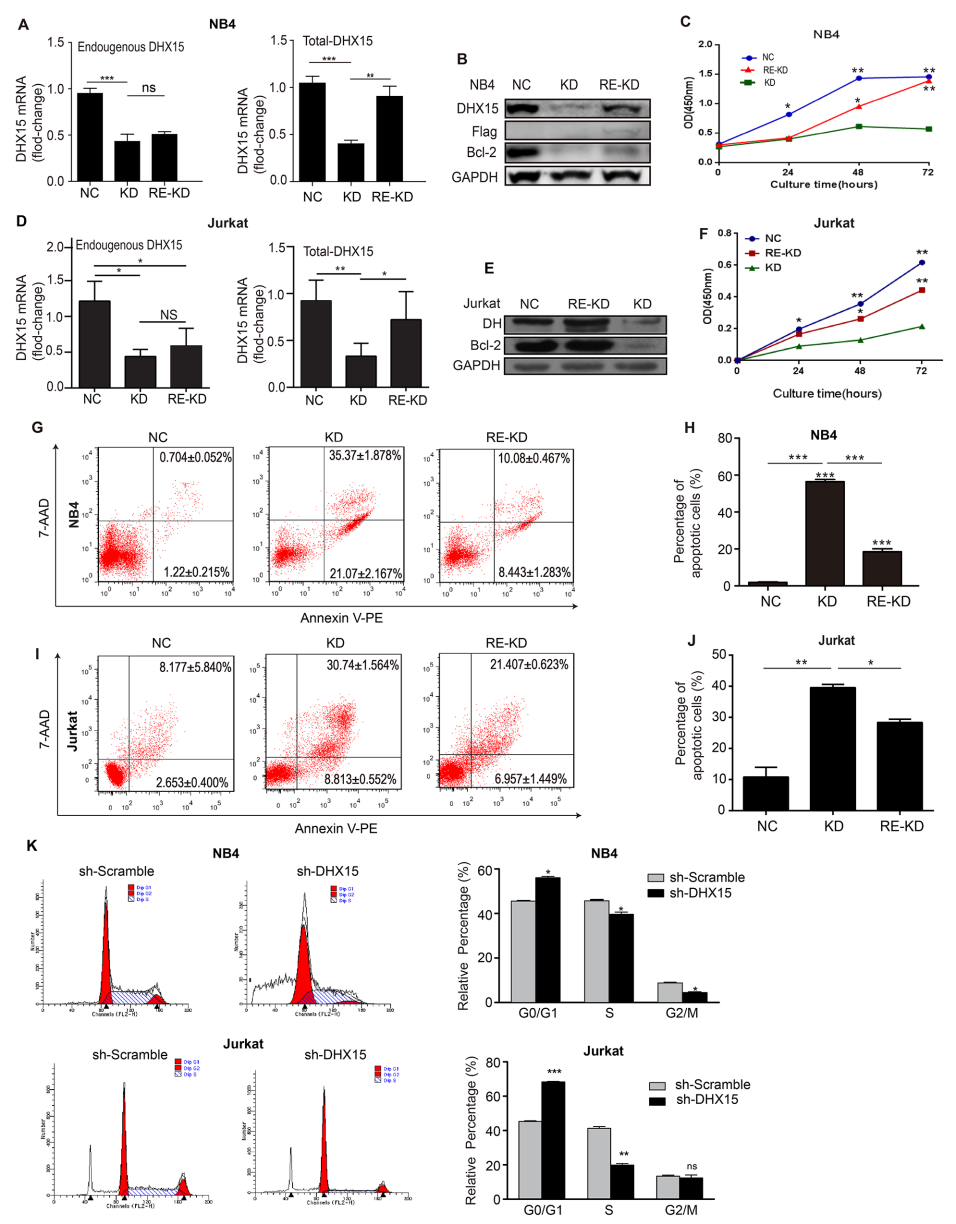
Silencing and restoration of *DHX15* **(A, B, D & E)** Real-time RT-PCR and western blots were used to validate the knockdown and restoration of the expression of *DHX15*. **(C & F)** CCK-8 analysis of NB4 cells and Jurkat cells. **(G-J)** Apoptosis analysis of NB4 cells and Jurkat cells. **(K)** Cell cycle analysis of NB4 cells and Jurkat cells. KD: knockdown, RE-KD: knockdown followed by restoration, NC: negative control, the scrambled shRNA group. ^*^P<0.05, ^**^P<0.01, ^***^P<0.001.

An expression data set of 1036 tumor cell lines of all types from the Broad Institute database showed increased expression levels of *DHX15* in leukemia cell lines compared with other tumor cell lines (Supplementary Figure 3). In addition, our experiments also showed higher expression levels of *DHX15* in leukemia cell lines (Supplementary Figure 4), especially in the NB4 and Jurkat cell lines. Therefore, we silenced and restored the expression of *DHX15* in the leukemia cell lines NB4 and Jurkat. Real-time PCR and western blot analysis showed that the *DHX15*-specific shRNA (sh*DHX15*#1) significantly suppressed the expression of *DHX15* by ~70% compared with a scrambled shRNA construct (Figure 4A, 4B, 4D and 4E). *DHX15* knockdown led to significant decreases in the number of viable NB4 and Jurkat cells measured by CCK-8 assay (Figure 4C and 4F) and in the number of colonies (almost by 100%) measured by a colony formation assay (Supplementary Figure 5). We then examined how down-regulation of *DHX15* decreased cell proliferation using FACS analysis. The results showed that knockdown of *DHX15* expression markedly induced cell apoptosis (Figure 4G-4J) and blocked the cell cycle transition from the G1 to S phase in NB4 and Jurkat cells (Figure 4K), indicating the induction of G1 arrest. In addition, we demonstrated that knockdown of *DHX15* expression by different shRNA sequences (sh*DHX15*#2) also significantly suppressed growth in NB4 cells, indicating no off-target effects (Supplementary Figure 6).

To confirm whether the restoration of *DHX15* expression can reverse the inhibition of cell growth in *DHX15*-silenced cells, we introduced exogenous *DHX15* expression into NB4 and Jurkat cells that were previously infected with lentivirus carrying sh*DHX15*#1. The results showed that the proliferation ability of NB4 and Jurkat cells was partly restored (Figure 4C and 4F), and the percentage of apoptotic cells was significantly reduced when *DHX15* expression was restored (Figure 4G-4J).

**Figure 5:**
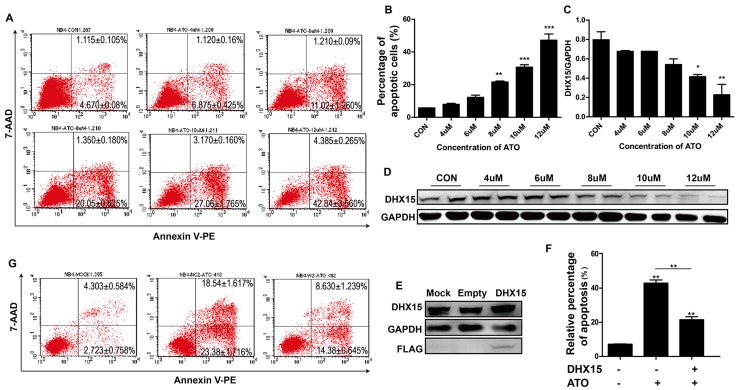
Overexpression of *DHX15* inhibited cell apoptosis **(A-B)** Apoptosis analysis of NB4 cells with ATO treatment at different concentrations showed a dose-dependent increase in the proportion of apoptotic cells. **(C-D)** Expression analysis of DHX15 in NB4 cells with ATO treatment at different concentrations showed a dose-dependent decrease in the expression of DHX15 with cell apoptosis. **(E)** Validation of the overexpression of DHX15 in NB4 cells. **(F-G)** Apoptotic analysis of NB4 cells with DHX15 overexpression and ATO treatment (12 μM) showed resistance to apoptosis. ^*^P<0.05, ^**^P<0.01, ^***^P<0.001.

To further explore the effect of *DHX15* expression on cell apoptosis, we treated NB4 cells with ATO, an apoptosis inducer, and found that *DHX15* was down-regulated with cell apoptosis (Figure 5A-5D). In addition, overexpression of *DHX15* inhibited ATO-induced cell apoptosis (Figure 5E and 5F).

### DHX15 regulates leukemia cell apoptosis through the NF-kB signaling pathway

**Figure 6:**
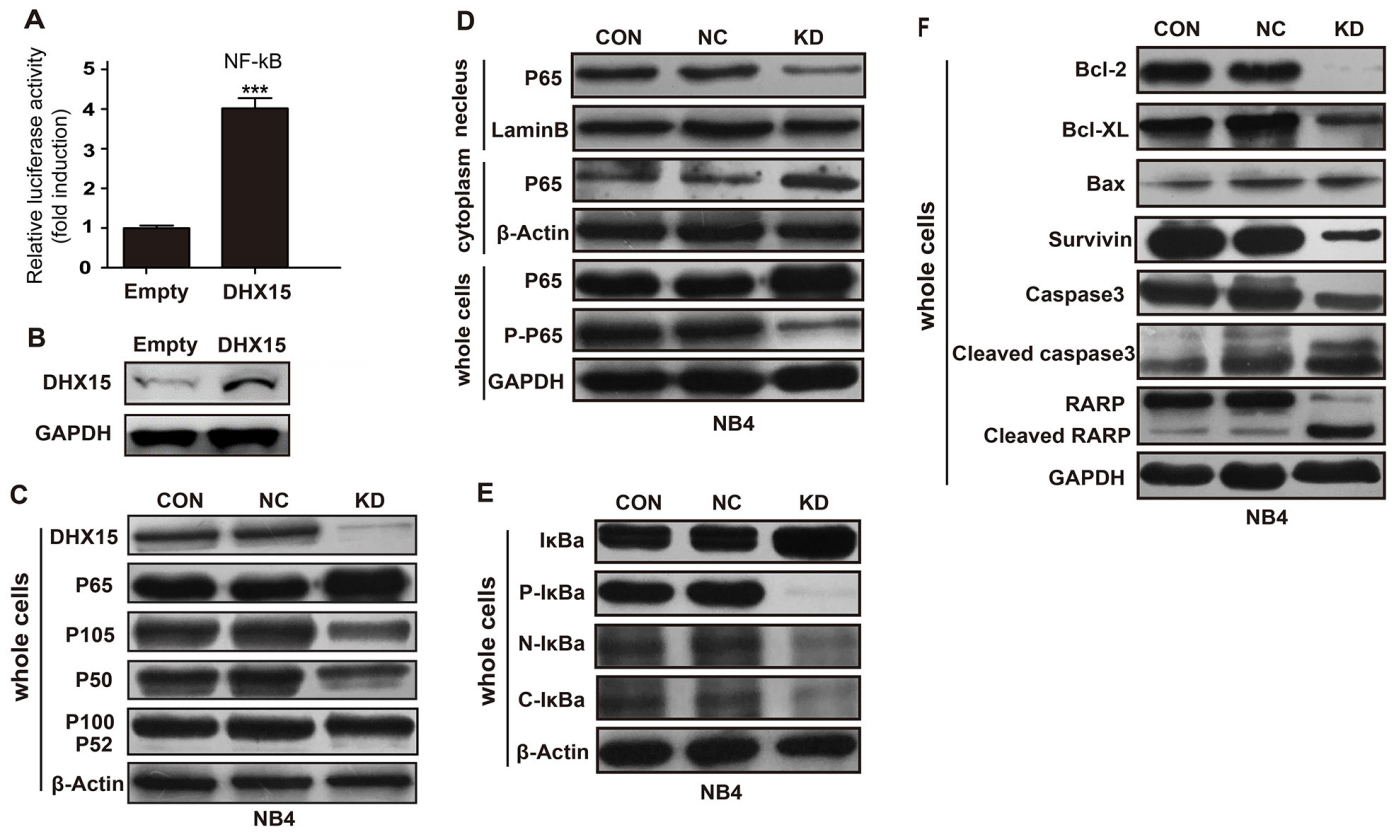
Effects of *DHX15* silencing on the NF-kB signaling pathway **(A-B)** Overexpression of *DHX15* activated *NF-kB* transcription. **(C)** Effects of *DHX15* knockdown on the *NF-kB* subunits. **(D)** Effects of *DHX15* knockdown on the nuclear translocation and activation of P65. **(E)** Effects of *DHX15* knockdown on the phosphorylation of IkBa and its dissociation with P65. **(F)** Effects of *DHX15* knockdown on the downstream targets of the *NF-kB* pathway. CON: blank (no virus), NC: negative control, KD: knockdown, Caspase 3: encoded by the *CASP3* gene.

*DHX15* has been implicated in antiviral immune responses through the regulation of the NF-kappaB signaling pathway;[14, 15] we, therefore, explored whether *DHX15* participates in leukemia cell proliferation and apoptosis through the *NF-kB* pathway. We performed an *NF-kB* luciferase assay and found that overexpression of *DHX15* activated *NF-kB* transcription (Figure 6A and 6B). Knockdown of *DHX15* reduced the expression level of the *NF-kB* subunit p105/p50 and increased the expression of the subunit P65 (Figure 6C); no change in expression of other *NF-kB* subunits was observed. Further detection showed decreased expression of P65 in the nucleus and increased expression of P65 in the cytoplasm (Figure 6D). In addition, the phosphorylated form of P65 (activated form) was reduced (Figure 6D). These results suggested that the nuclear translocation and activation of P65 were inhibited. We then detected the expression of P65's inhibitor, IkBa, and found that the phosphorylated form of IkBa was reduced (Figure 6E), which meant that its dissociation with P65 and subsequent degradation were reduced and that the nuclear translocation and activation of P65 would be inhibited, which was consistent with previous results.

Moreover, we found that knockdown of *DHX15* suppressed the expression of multiple targets of the *NF-kB* pathway (Figure 6F), including BCL2, BCL-XL and SURVIVIN, while BAX was not affected. In addition, knockdown of DHX15 activated CASP3 and PARP (Figure 6F).

## DISCUSSION

This is the first study to investigate the role of *DHX15*, a member of the DEAH-box RNA helicase family, in the pathogenesis and progression of leukemia. *DHX15* has been reported to be ubiquitously expressed in several tumor cell lines and multiple normal tissues and organs.[6] Our alignment analysis of 11 species showed that human *DHX15* was a highly conserved protein, sharing 99%, 83% and 80% identities of the amino acid sequence with mouse, zebrafish, and yeast, respectively. The ubiquitous expression and evolutionary conservation of *DHX15* suggest that it may serve important functions and be required for life. Moreover, we found a distinct expression pattern of *DHX15*. It was highly expressed in the early phase of stem cells, including ESCs and HSCs in mice, and then appeared to decrease as the cells differentiated to more mature stages (CMPs and GMPs), reaching its lowest expression level in the terminal differentiation stages (granulocytes and macrophages). This was consistent with our results that extremely low expression of *DHX15* was detected in human blood samples from healthy controls, which mainly consisted of mature blood cells. In contrast, *DHX15* was commonly over-expressed in AML patients and leukemia cell lines. In addition, *DHX15* was down-regulated when patients achieved disease remission or when leukemia cell lines were induced to differentiate. In addition, recurrent mutations and copy number gains of *DHX15* were found in solid tumors or hematopoietic malignancies. These results suggest that the expression of *DHX15* should be strictly regulated and that abnormal expression or inappropriate activation of *DHX15* caused by any reason may contribute to carcinogenesis.

*DHX15* knockdown greatly inhibited cell proliferation, induced cell apoptosis, and led to G1 arrest in leukemia cells. We used two shRNAs targeting different regions of *DHX15* in independent assays and observed similar results, which excluded the possibility of off-target effects. Moreover, the restoration of *DHX15* expression reversed the inhibition of cell growth and reduced cell apoptosis, which further excluded the possibility of off-target effects and suggested a strong effect of *DHX15* on cell proliferation and apoptosis regulation.

*DHX15* was down-regulated when patients who over-expressed *DHX15* achieved disease remission, which suggested that *DHX15* overexpression occurred mainly in leukemia cells and may be used for minimal residual disease (MRD) detection. Moreover, we found that overexpression of *DHX15* was associated with poor OS and RFS and conferred an even worse OS in the group with poor cytogenetic prognostics. Multivariate analysis demonstrated that *DHX15* overexpression was an unfavorable independent factor associated with OS in AML. Taken together, these results suggest that *DHX15* can help to achieve a more sophisticated stratification for AML patients, especially in the poor cytogenetics group.

*DHX15* was down-regulated with leukemia cell apoptosis, and overexpression of *DHX15* partially blocked ATO-induced cell apoptosis. These data indicated that *DHX15* may participate in ATO-induced cell apoptosis, and its overexpression may be related to ATO-resistance in acute promyelocytic leukemia (APL) patients.

Several studies suggest that *DHX15* participates in RNA virus-induced apoptosis by facilitating the *MAVS*-mediated activation of *NF-kB* and MAPK signaling.[14, 15] In our study, we confirmed that the overexpression of *DHX15* activated *NF-kB* transcription. Knockdown of *DHX15* reduced the expression level of the *NF-kB* subunit P105/P50 and inhibited the nuclear translocation and activation of P65 by suppressing the phosphorylation of its inhibitor, IkBa, which thus inhibited the downstream signaling transduction of *NF-kB*. Consistent with this result, we found that multiple targets of the *NF-kB* pathway were down-regulated when *DHX15* was knocked down, including BCL-2, BCL-XL and SURVIVIN. It has been reported that the down-regulation of BCL-2 and BCL-XL promotes cell apoptosis by activating CASP3 and PARP.[16] Interestingly, we found that the knockdown of *DHX15* activated *CASP3* and *PARP*. Therefore, we suggest that *DHX15* participates in the regulation of leukemia cell growth and apoptosis through the *NF-kB* –*BCL-2/BCL-XL* –*CASP3*/*PARP* pathway.

Together, we reported a recurrent mutation in DHX15 in both familial and sporadic AML patients and found a distinctive expression pattern of DHX15 in hematopoietic cells. Silencing DHX15 greatly inhibited leukemia cell proliferation and induced cell apoptosis and G1-phase arrest. The restoration of DHX15 expression rescued cell viability and reduced cell apoptosis. In addition, overexpression of DHX15 was associated with poor OS and RFS and conferred an even worse OS in the group with poor cytogenetic prognostics. These results suggest that DHX15 may contribute to leukemogenesis and would be a promising marker for AML diagnosis, prognosis and MRD detection. We preliminarily found that DHX15 participates in the regulation of leukemia cell apoptosis through the NF-kB-BCL-2/BCL-XL-CASP3/PARP pathway; however, further studies are required to clarify whether abnormal RNA helicase activity or splicing is the mechanism underlying the multiple functions of DHX15.

## MATERIALS AND METHODS

### Tissue samples and cell lines

This study was approved by the Expert Committee of Fujian Medical University Union Hospital in China (equivalent to an institutional review board). Informed consent was obtained from all of the examined subjects in accordance with the Declaration of Helsinki. We previously reported an AML-predisposed family with 11 cases in four generations (Supplementary Figure 1) [17], and samples from one of the patients, III-15, before and after his disease onset were obtained. DNA samples from 240 sporadic de novo AML patients and 508 healthy individuals were also obtained. RNA samples and clinical information were obtained from 135 AML patients and 84 healthy individuals. Peripheral blood samples obtained during remission were available from the sporadic patients who overexpressed *DHX15*. NB4 and Jurkat cells were grown in RPMI-1640 medium supplemented with 10% fetal bovine serum (GIBCO), 100 U/ml penicillin and 100 μg/ml streptomycin (Invitrogen) and at 37°C in 5% CO_2_.

### Sequencing, genotyping and bioinformatic analysis

Whole exome sequencing (WES) was performed on samples from patient III-15 before and after his AML onset. An in-house pipeline was used to analyze WES data and to identify the somatic mutations of III-15. Somatic mutations of III-15 were defined as mutations that were present in the samples at his AML onset but absent in the samples before his AML onset. We chose the two bases with the highest frequency at each genomic locus from data set of III-15's disease onset and then compared the frequency distribution of the two bases between the exome data sets gained before and after III-15's AML onset using Fisher's exact test. The somatic mutations of III-15 were defined as variants that had significant differences (P1e-8) at the base frequency distribution, and the reads supporting the variants were fewer than two in data set from III-15 before his AML onset. All the somatic mutations were further validated in the corresponding samples by Sanger sequencing and screened in 240 sporadic AML patients and 508 healthy controls using SNaPshot technology (See Supplementary Materials and Supplementary Table 1) [18]. Recurrent mutations found in SNaPshot genotyping were again validated by Sanger sequencing.

### Expression analysis of DHX15 in AML patients, healthy controls, leukemia cell lines, and hematopoietic cells

Quantitative real-time PCR was used to analyze the expression level of *DHX15* in 135 de novo AML patients, 84 normal controls, and 30 paired samples from patients at their diagnosis of AML and disease remission phase (See Supplementary Materials). Western blotting was used to detect the expression of *DHX15* in 7 AML patients, 4 normal controls, 6 paired samples and 7 leukemia cell lines. In addition, data mining of *DHX15* expression in cancer cell lines and stem cell lines was accomplished using data from the Broad Institute database (http://www.broadinstitute.org) and the EMBL database (http://www.ebi.ac.uk).

### Induction of leukemia cell differentiation

Granulocytic differentiation in NB4 cells was induced with ATRA (Sigma-Aldrich). Morphology was evaluated by conventional light-field microscopy to examine May-Grünwald-Giemsa-stained cytospins using an Olympus BX51 (Japan) optical microscope, and the CD11b levels were determined using the PE-CD11b antibody (BioLegend, #301305) and a FACSAria Flow Cytometer (BD Biosciences). Real-time PCR and western blots were used to analyze the expression level of DHX15 in NB4 cells with and without ATRA treatment.

### Silence and restoration of DHX15 expression

A lentivirus vector (Mu6-MCS-Ubi-EGFP) expressing human DHX15-shRNA was constructed, and the targeted sequences were as follows: shDHX15#1 (TGGTTCGATAATGGCCTTT), shDHX15#2(TGTTC TAATGAGGTCCTAT), and shDHX15#3(TAAGAGAA TAAAGCGTGAA). NB4 and Jurkat cells were infected with DHX15 shRNA or the scrambled vector at a MOI of 50. DHX15 sequences were cloned into lentivirus-based expression vectors (Ubi-MCS-3FLAG-SV40-EGFP). For the knockdown assay, NB4 and Jurkat cells were infected with a lentiviral vector carrying *DHX15* shRNA or the scrambled sequence at a MOI of 50. For the restoration assay, NB4 and Jurkat cells were first infected with *DHX15* shRNA or scrambled shRNA at a MOI of 50, followed by compulsory overexpression of exogenous *DHX15* using lentivirus-based expression vectors at a MOI of 100 one day later. Here, we used the shDHX15#1 sequence, which targeted the 3’UTR of DHX15, so that the exogenous expression of DHX15 (not containing the 3’UTR of DHX15) would not be knockdown by shDHX15#1. The silencing and restoration efficiency of *DHX15* were confirmed by western blots and qRT-PCR. Two sets of primers were used to validate the endogenous (primers located in the 3’UTR) and total expression levels (primers located in coding region) of DHX15. Six hours after infection, cell viability was assessed by Cell Counting Kit 8 (CCK8) according to the manufacturer's instructions (Dojindo Molecular Laboratories, Kumamoto, Japan). For clonogenic assays, cells were seeded in 24-well plates at 200 cells/well. In addition, the clones were counted after one week using light-field microscopy. For cell cycle analysis, cells were stained with propidium iodide (Sigma), and the percentages of cells in the subG0/G1, S and G2/M phases were determined with a FACSCalibur Flow Cytometer (BD Biosciences). For apoptosis analysis, the cells were stained with Annexin V-PE and 7-AAD for 15 min at room temperature in the dark and then analyzed with a FACSCalibur Flow Cytometer (BD Biosciences). In addition, the changes in the expression levels of cell cycle- or apoptosis-related proteins were analyzed in DHX15 knockdown cells using western blotting.

### ATO-induced apoptosis assay

NB4 cells were treated with the apoptosis inducer ATO (arsentic trioxide) (Amresco) at a wide range of concentrations (0, 4 μM, 6 μM, 8 μM, 10 μM, 12 μM), and the proportion of apoptotic cells and the expression levels of *DHX15* were detected 48 hours later using the same methods described above. To further estimate the effect of *DHX15* expression on apoptosis in leukemia cells, we overexpressed *DHX15* in NB4 cells and treated them with ATO at a concentration of 12 μM.

### Luciferase assay

293T cells seeded in 24-well plates were transiently cotransfected with 50 ng of NF-kB luciferase and 50 ng of Renilla luciferase reporter vectors plus 300 ng of the wild-type DHX15 expression vector or the empty control vector pCMV [provided by the School of Life Sciences, Fudan University]. The P65 promoter was linked with the luciferase reporter. At 24 h posttransfection, cells were collected and lysed with 1X lysis buffer. The luciferase activity in the total cell lysate was detected with the Dual-Luciferase Reporter Assay (Promega Madison, WI) according to the manufacturer´s instructions.

### Statistical analyses

The *DHX15* gene was considered overexpressed if its expression value was higher than the cut-off value (mean±3 s.d.) defined by the analysis of 84 healthy controls.[19] All statistical analyses were performed using SPSS 20 (SPSS Inc, Chicago, Illinois) (See Supplementary Materials).

## SUPPLEMENTARY MATERIALS FIGURES AND TABLES


