# Supplementary material for: DHX15 is associated with poor prognosis in acute myeloid leukemia (AML) and regulates cell apoptosis via the NF-kB signaling pathway
Source: Oncotarget. 2017 Aug 16;8(52):89643–54. doi: 10.18632/oncotarget.20288 (PMC5685698; doi:10.18632/oncotarget.20288)
Supplement: Supplementary file 1 [file oncotarget-08-89643-s001.pdf]

DHX15 is associated with poor prognosis in acute myeloid leukemia (AML) and regulates cell apoptosis via the NF-kB signaling pathway

SUPPLEMENTARY MATERIALS

Tissue samples and cell lines

All patients were diagnosed according to WHO classifications [2] and were treated with standard procedures approved by Fujian Institute of Hematology based on the NCCN guideline 2008 for AML. Among them, RNA samples and clinical information were obtained from 135 AML patients and 84 healthy individuals. Peripheral-blood samples obtained during remission were available from the sporadic patients who overexpressed DHX15. Cell line NB4 was kindly provided by Dr Jinghan Wang (School of Medicine, Shanghai Jiao Tong University, Shanghai, China) in 2013. Jurkat cell was purchase from the Chinese Academy of Sciences in Shanghai. Cell lines were authenticated by analysis of their genetic alterations.

Whole exome sequencing (WES) and bioinformatics analysis

A WES was performed on samples gained from patient III-15 before and after his AML onset. Briefly, 15mcg of genomic DNA from each sample was enriched for the target region of the consensus coding sequence (CCDS) exons with NimbleGen 2.1M human exome array and subsequently sequenced on the Illumina Hiseq2000 platform following the manufacturer's instructions (Illumina, CA). [3] The raw data was mapped to the human genome reference sequence (hg19) with Burrows-Wheeler Aligner (BWA). [4] Single nucleotide variants (SNV) and short Insertion/Deletion (InDels) were detected with SOAPsnp [5] and SAMtools, [6] respectively. After that, the low-quality variations were filtered out using the following criteria: (i) quality score ≧20 (Q20); (ii) average copy number at the allele site ≦2; (iii) distance of two adjacent SNPs ≧5bp; and (iv) sequencing depth ≧4 and ≦1000. Then we used ANNOVAR7 to annotate the confident variant results.

An in-house pipeline was used to identify the somatic mutations of III-15, which were defined as mutations that were presented in samples at his AML onset while absent in samples before his AML onset. We picked out the two bases of highest frequency at each genomic loci from data set gained at III-15's disease onset, and then compared the frequency distribution of the two bases between exome data sets gained before and after III-15's AML onset using Fisher's exact test. The somatic mutations of III-15 were defined as variants which have significant difference (P≦1e-8) at base frequency distribution and the reads supporting the variants were fewer than two in data set from III-15 before his AML onset.

Quantitative real-time PCR

Total RNA was isolated using TRIzol Reagent (Invitrogen) and 1ug of RNA was reverse transcribed into cDNA using SuperScript III First Strand Synthesis (Invitrogen) according to manufacturer's recommendations and used for gene expression analyses. For qPCR analysis, the SYBR Green dye (Takara) and ABI Prism 7900 Sequence Detection System were used (both from Life Technologies). All samples were run in triplicate. Relative quantification was done using the 2-ΔCt method (ΔCT = (CT DHX15 – CT β-actin), normalizing to Actin. Primers used for qPCR analysis are: DHX15: F: 5-TCTACACTTCCACCTCAGCAGCA-3; R: 5-CCAGG ATCAATCACAAACACCACAC-3; ACTIN: F:5-AGCC TCGCCTTTGCCGA-3; R: 5-GCGCGGCGATATCAT CATC-3.

Western-blot

Cells were washed in PBS and lysed using RIPA lyses buffer (Cell Signaling, Boston, MA). Cell lysates were separated by gradient (4–12%) SDS-polyacrylamide gel electrophoresis and transferred to PVDF membranes. Membranes were probed with the appropriate antibodies and visualized using standard techniques. As loading controls we used anti-actin or anti-GAPDH antibodies. Anti-DHX15 (12265-1-AP Rabbit polyclonal antibody) and anti-GAPDH (Ab103-03 mouse monoclonal antibody) were perchused from Proteintech and Vazyme Biotech, respectively. Anti -bcl2 (Ab32124), anti-p105/p50(ab32360), anti-IKBα(ab32518), anti-p-IKBα(ab133462), anti-Rel-B(ab33917), anti-p100/p52(ab109440), and anti-Lamin B1(ab133741) were purchased from Abcam (Cambridge, UK). Anti- PARP(9532T), anti-p65(8242T), anti-p-p65(3033T), anti-caspase3(9665T), anti-cleaved-caspase3(9664T), anti-bcl-2(4223S), anti-bax(5023T), anti-bcl-xl(2764T), anti-IKBα(4814T, N-terminal), and anti-IKBα(9247S, C-terminal) were purchased from Cell Signaling Technology.

Statistical analyses

DHX15 gene was considered overexpressed if its expression value was higher than the cut-off value (mean±3 s.d.), defined by the analysis of 84 healthy controls.[7] The possible association between DHX15 expression and other biological features was analyzed using the X2-test or the Fisher's exact test, as appropriate, for categorical variables, and the Mann–Whitney test for continuous variables. Definition of complete remission (CR) and survival end points such as OS and RFS followed the recommended consensus criteria.[8] The Kaplan-Meier method and the log-rank test were used to calculate distribution estimation and survival distributions of OS and RFS. The role of each variable on obtainment of complete remission (CR) after induction therapy was calculated by univariate and multivariate logistic regression. A Cox proportional hazards model was constructed for survival end points. Characteristics selected for inclusion in the multivariate analysis were those for which there was some indication of a significant association in univariate analysis ($P \leq 0.2$). Characteristics selected for inclusion in the multivariate analysis were age, white blood count, subtype, cytogenetics, FLT3-ITD and NPM1 mutations, and DHX15 high/low expression status. DHX15 expression was transformed in categorical variable for survival analysis using the cut-off (mean+3 s.d.). Hazard ratios (HR) and 95% confidence interval (CI) were obtained for each significant prognostic factor. All statistical analyses were performed using SPSS 20 for Windows (SPSS Inc, Chicago Illinois). P values less than 0.05 were considered statistically significant. Mechanistic and biological experiments were analyzed with paired and unpaired 2-tailed t tests as required, and a P value of less than 0.05 was considered significant. Data are presented as mean ± SEM in all figures where error bars are shown.

REFERENCES

1. Pan LL, Huang YM, Wang M, Zhuang XE, Luo DF, Guo SC, Zhang ZS, Huang Q, Lin SL, Wang SY. Positional cloning and next-generation sequencing identified a TGM6 mutation in a large Chinese pedigree with acute myeloid leukaemia. Eur J Hum Genet. 2015; 23: 218-23. https://doi.org/10.1038/ejhg.2014.67.
2. Vardiman JW, Thiele J, Arber DA, Brunning RD, Borowitz MJ, Porwit A, Harris NL, Le Beau MM, Hellstrom-Lindberg E, Tefferi A, Bloomfield CD. The 2008 revision of the World Health Organization (WHO) classification of myeloid neoplasms and acute leukemia: rationale and important changes. Blood. 2009; 114: 937-51. https://doi.org/10.1182/blood-2009-03-209262.
3. Wang JL, Yang X, Xia K, Hu ZM, Weng L, Jin X, Jiang H, Zhang P, Shen L, Guo JF, Li N, Li YR, Lei LF, et al. TGM6 identified as a novel causative gene of spinocerebellar ataxias using exome sequencing. Brain. 2010; 133: 3510-8. https://doi.org/10.1093/brain/awq323.
4. Sunyaev S, Ramensky V, Koch I, Lathe W 3rd, Kondrashov AS, Bork P. Prediction of deleterious human alleles. Hum Mol Genet. 2001; 10: 591-7.
5. Li R, Li Y, Fang X, Yang H, Wang J, Kristiansen K. SNP detection for massively parallel whole-genome resequencing. Genome Res. 2009; 19: 1124-32. https://doi.org/10.1101/gr.088013.108.
6. Li H, Handsaker B, Wysoker A, Fennell T, Ruan J, Homer N, Marth G, Abecasis G, Durbin R. The Sequence Alignment/Map format and SAMtools. Bioinformatics. 2009; 25: 2078-9. https://doi.org/10.1093/bioinformatics/btp352.
7. Vicente C, Vazquez I, Conchillo A, Garcia-Sanchez MA, Marcotegui N, Fuster O, Gonzalez M, Calasanz MJ, Lahortiga I, Odero MD. Overexpression of GATA2 predicts an adverse prognosis for patients with acute myeloid leukemia and it is associated with distinct molecular abnormalities. Leukemia. 2012; 26: 550-4. https://doi.org/10.1038/leu.2011.235.
8. Dohner H, Estey EH, Amadori S, Appelbaum FR, Buchner T, Burnett AK, Dombret H, Fenaux P, Grimwade D, Larson RA, Lo-Coco F, Naoe T, Niederwieser D, et al. Diagnosis and management of acute myeloid leukemia in adults: recommendations from an international expert panel, on behalf of the European LeukemiaNet. Blood. 2010; 115: 453-74. https://doi.org/10.1182/blood-2009-07-235358.

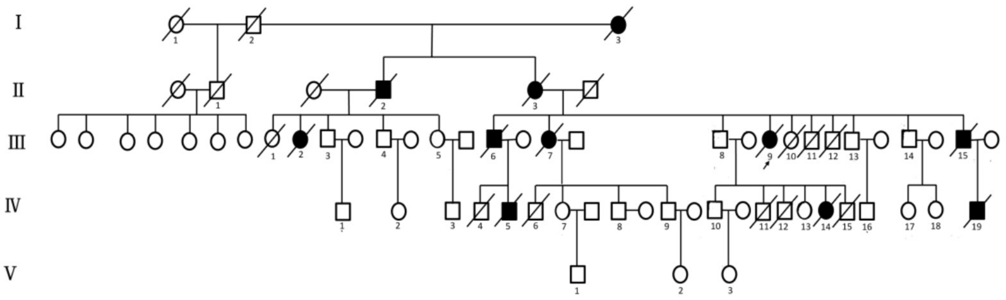

Supplementary Figure 1: Familial AML pedigree.[1] Squares represent males; circles represent females. The index patient is indicated by an arrow. Deceased subjects are indicated by a diagonal line. Roman numerals denote generations. Arabic numerals indicate the position within the same generation. Open symbols represent unaffected individuals; closed symbols represent affected individuals. Closed symbols with a diagonal line indicate that the individual died from AML

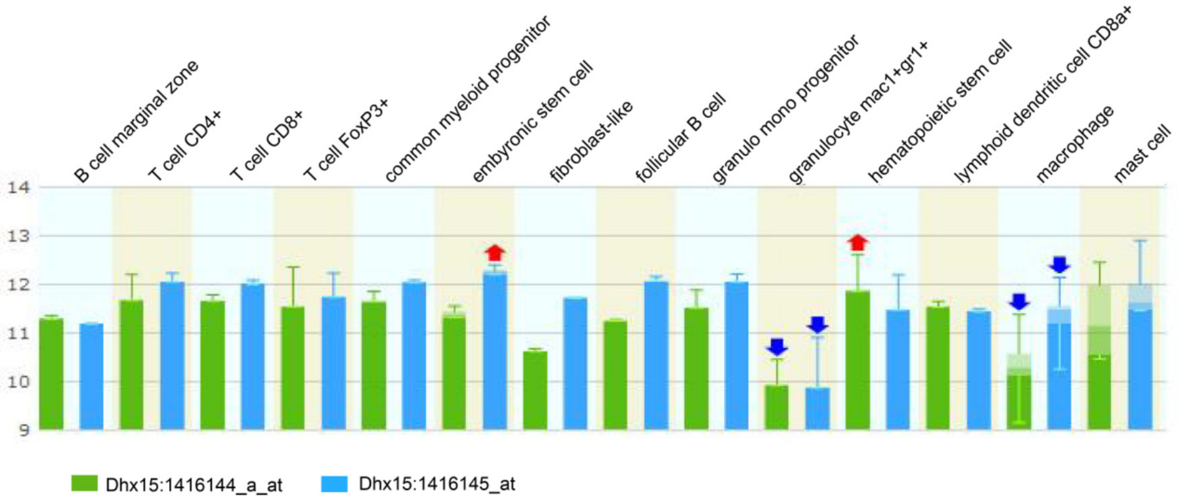

Supplementary Figure 2: DHX15 expression in different phases of hemapoietic stem/progenitor cells. This gene expression microarray data was gained from EMBL database (http://www.ebi.ac.uk). Blue bars and green bars represented two independent sets of probes that detect the expression level of DHX15. Each bar was presented as mean±SEM of three independent experiments. And DHX15 expression showed a higher level in early phase of stem cell including ESCs and HSCs, and lower level in granulocyte and macrophage (data gained from EMBL database).

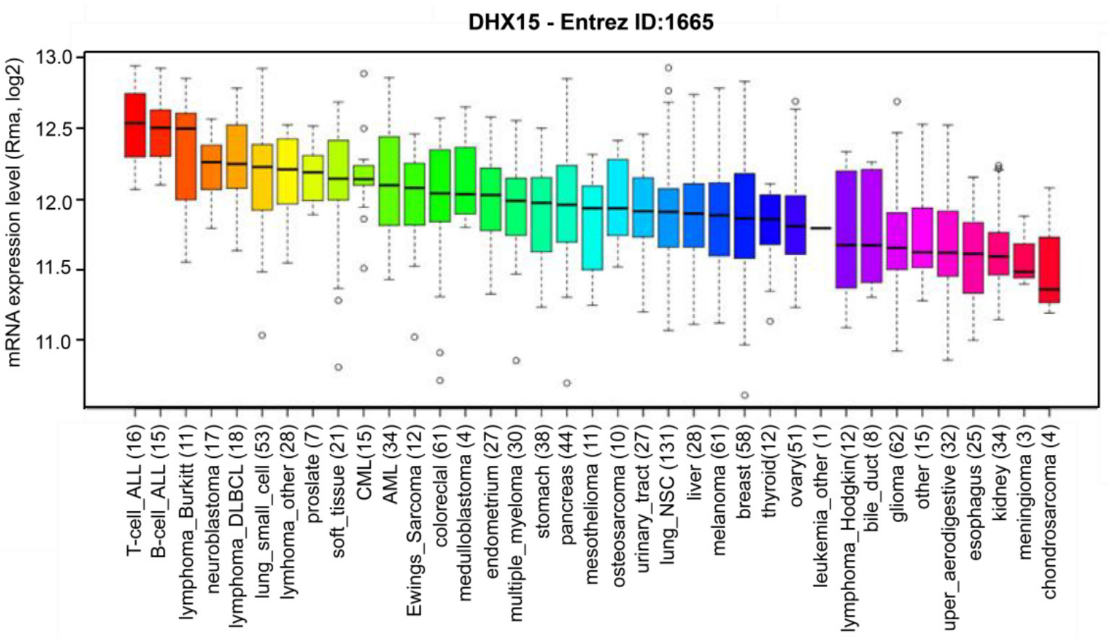

Supplementary Figure 3: DHX15 expression in 1036 tumor cell lines of all types in Broadinstitute. X-axis represents the classifications of tumor cell lines, and the Arabic number in bracket means the number of types of cell lines included in this classification. The order of the types of cell lines listed was according to their expression level of DHX15, from high to low. Y-axis represents the expression level of mRNA in the specific classification of tumor cell line. DHX15 was relevantly elevated in leukemia cell lines comparing with other tumor cell lines.

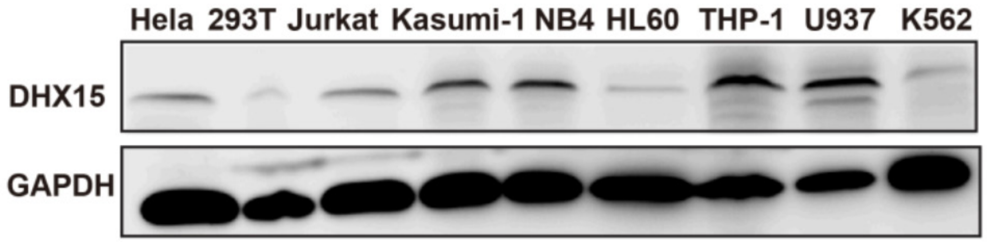

Supplementary Figure 4: Western-blotting analysis of expression level of DHX15 in leukemia cell lines. DHX15 was relevantly elevated in NB4 and Jurkat cell lines.

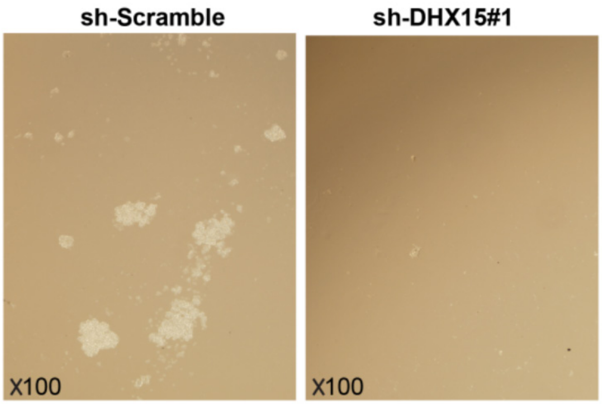

Supplementary Figure 5: DHX15 knock-down inhibited colony formation of NB4 cells.

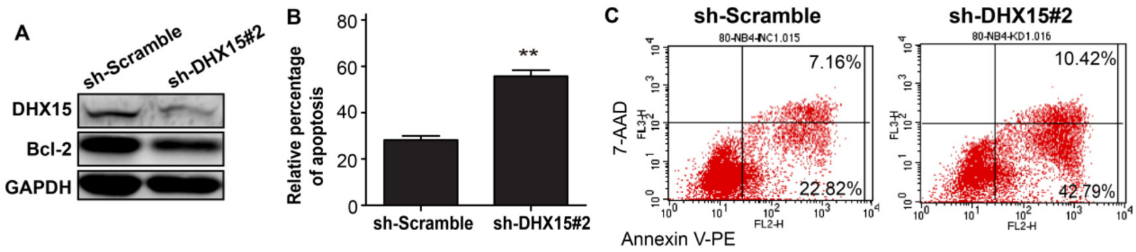

Supplementary Figure 6: Knock-down effect of shDHX15#2 on NB4 cell apoptosis. (A) Validation of knock-down effects in NB4 cell line using western-blots. **(B-C)** Apoptotic analysis of NB4 cells.

Supplementary Table 1: List of primers used in SNaPshot

Gene	PCR reaction	Genotyping reaction
DHX15	F: 5-TGGATGACATAAGCCTGCACTAGC<br>R: 5-GTGTGGAGTACATGCGATCATTACC	5-TTTTTTTTGGCCAGGAA GTTGGTTACTCCATT
EP300	F: 5-AATGTCCTTGTCGCCGTTGATGA<br>R: 5-GGTAGCCACCAGACACAGATAGC	5-TTTTTTTTTTTTTTTTGATGACCCT TCCCAGCCTCAAAC
SSH2	F: 5-GACCCATCTCTTAAAGTCCCTCTGT<br>R: 5-GAATCTAATGCTGCCACTGATCTGAC	5-TTTTTTTTTTTTTTTTCTTTCTGTTC GTTCACGTTCAGTAGG
MIR142	F: 5-AGCAGTGGCGTGATCTCCGAA<br>R: 5-CCTAGTCTCTACCTGAGTGTCTCTGAA	5-TTTTTTTTTTTTTTTTTTTTTTGCACT ACTAACAGCACTGGAGGGTGTAG
ANXA11	F: 5-CTTGCCGTACATCCGCTTATACTCTG<br>R: 5-TGCTCTTGGGTGGACTCTCTTTAGG	5-TTTTTTTTTTTTTTTTTTTTTTTTTTTTTTTTTTT TTTTTTTGTCCAGGAGGTCGGTCTCGCTG
CYB561D2	F: 5-GCTCACTCTGGTTCACTGCCTCT<br>R: 5-GACACATGATGCCTATGTTCCTCTGG	5-TTTTTTTTTTTTTTTTGTAGGCAT TGCTCACCTGGTTCATAA
HGS	F: 5-TTGTTCTGCTTGTCCCTTGCCTTC<br>R: 5-TCTCTCACACCCACCTGCCCTA	5-TTTTTTTGCTCGTAGCAGGGC TCACACA
MMP14	F: 5-GATCATCATTGAGGTGGACGAGGAG<br>R: 5-CAGAGTTCAGAGGTTAAGGTATGTCAGG	5-TTTTTTTTTTTTTTGCTGCCGTGG TGCTGCCC
MYO6	F: 5-CTCTCCGTCTCTGTCTCTGTCTCTG<br>R: 5-AAGCCTTCGTCGTCTCTGATATTCCTA	5-TTTTTTTTTTTTTTTGCCTTCGTCGTC TCTGATATTCCTATGA
FETUB	F: 5-TGTCTCTGTGACTTGTGACTTCTTTGA<br>R: 5-AAGTGGTGAGCAAGGTGGTTGG	5-TTTTTTTTTTTTTTTTTTAGACAG GCAATTAGATTGAAAAATACCTGT
KIF21A	F: 5-CGAGTAACTTTCCCAGCCACTTTATCT<br>R: 5-GCTTTGTTTCTTCCTGTTTGTACCTCA	5-TTTTTTTTTTTTTTTTTTTTTTTG CTTACTGTACCTCTTCAGTTTTGCGA
SEPX1	F: 5-ATTGCCACACTTGCCACAGGAC<br>R: 5-GTTCTCCAGCCGCTCGAAGTATG	5-TTGGCGTGAATGGTCTCGGTGAAC
ROS1	F: 5-GCTTCGACATTCAACCAGTCCTCTT<br>R: 5-AAGTTCCACATCACCGTGTCACAT	5-GGCTCCACTTCCCAGCAAGAGA

Supplementary Table 2: List of acquired mutations of familial AML patient III-15

Gene	Chr	Pos	Ref Base	Diff Base	Function	AAChange
DHX15	4	24572314	G	C	nonsynonymous	NM_001358:c.664C>G:p.(R222G)
EP300	22	41556727	G	A	splicing	NM_001429:c.3671+1G>A
SSH2	17	27994241	T	C	exonic/splicing	NM_033389:c.729A>G:p.(I243M)
MIR142	17	56408623	A	G	ncRNA_exonic	NR_029683:n.57A>G
ANXA11	10	81917473	C	A	nonsynonymous	NM_001157:c.1382G>T:p.(R461L)
CYB561D2	3	50391122	A	G	nonsynonymous	NM_007022:c.616A>G:p.(I206V)
HGS	17	79658570	G	A	nonsynonymous	NM_004712:c.631G>A:p.(V211M)
MMP14	14	23315135	G	A	nonsynonymous	NM_004995:c.1636G>A:p.(V546M)
MYO6	6	76576268	T	C	nonsynonymous	NM_004999:c.1700T>C:p.(V567A)
FETUB	3	1.86E+08	C	T	nonsynonymous	NM_014375:c.776C>T:p.(S259L)
KIF21A	12	39727030	C	T	nonsynonymous	NM_001173463:c.2432G>A:p.(R811H)
SEPX1	16	1991328	G	A	nonsynonymous	NM_016332:c.134C>T:p.(A45V)
ROS1	6	1.18E+08	C	T	nonsynonymous	NM_002944:c.5843G>A:p.(R1948H)
